# Supplementary material for: Complex‐centric proteome profiling by SEC‐SWATH‐MS
Source: Mol Syst Biol. 2019 Jan 14;15(1):e8438. doi: 10.15252/msb.20188438 (PMC6346213; doi:10.15252/msb.20188438)
Supplement: Supplementary file 7 — Dataset EV6 [file MSB-15-e8438-s007.zip › feature_plots_bioplex/Q99571.pdf]

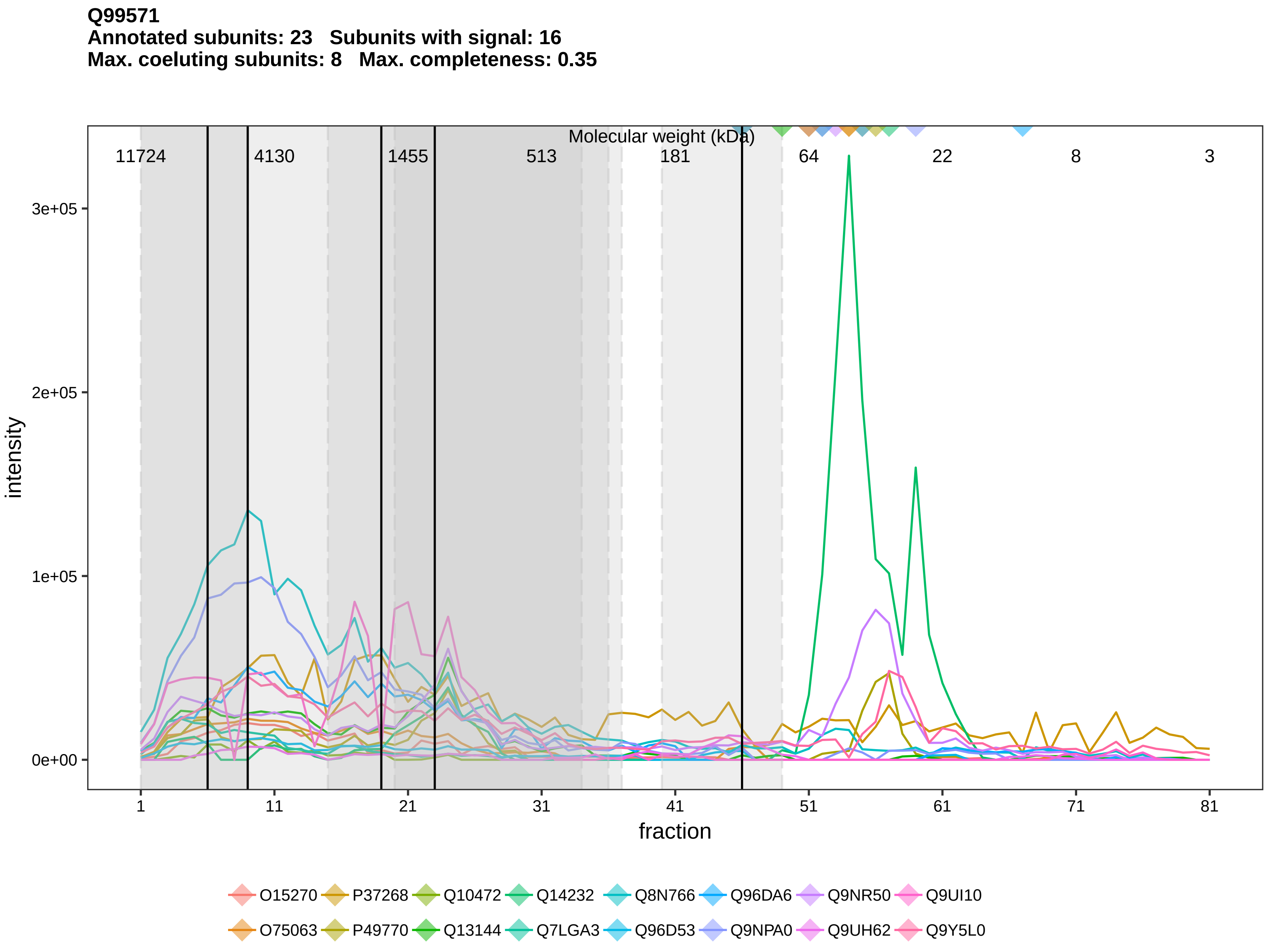

Q99571
Annotated subunits: 23 Subunits with signal: 16
Max. coeluting subunits: 8 Max. completeness: 0.35
Molecular weight (kDa)
11724
4130
1455
513
181
64
22
8
3
intensity
3e+05
2e+05
1e+05
0e+00
fraction
1
11
21
31
41
51
61
71
81
O15270
P37268
Q10472
Q14232
Q8N766
Q96DA6
Q9NR50
Q9UI10
O75063
P49770
Q13144
Q7LGA3
Q96D53
Q9NPA0
Q9UH62
Q9Y5L0
